# On-Demand Release of Anti-Infective Silver from a Novel Implant Coating Using High-Energy Focused Shock Waves

**DOI:** 10.3390/pharmaceutics15092179

**Published:** 2023-08-22

**Authors:** Jan Puetzler, Julian Hasselmann, Melanie Nonhoff, Manfred Fobker, Silke Niemann, Christoph Theil, Georg Gosheger, Martin Schulze

**Affiliations:** 1 Department of General Orthopedics and Tumor Orthopedics, Muenster University Hospital, Albert-Schweitzer-Campus 1, 48149 Muenster, Germany; 2 Materials Engineering Laboratory, Department of Mechanical Engineering, University of Applied Sciences Muenster, 48565 Steinfurt, Germany; 3 Central Laboratory, Muenster University Hospital, Albert-Schweitzer-Campus 1, 48149 Muenster, Germany; 4 Institute of Medical Microbiology, Muenster University Hospital, Domagkstraße 10, 48149 Muenster, Germany

**Keywords:** anti-infective silver, implant coating, implant-related infection, extracorporeal shock waves

## Abstract

Implant-related infections are a significant concern in orthopedic surgery. A novel anti-infective implant coating made of bioresorbable polymer with silver nitrate was developed. A controlled release of silver ions into the vicinity of the prosthesis can be triggered on-demand by extracorporeal shock waves to effectively combat all clinically relevant microorganisms. Microscopy techniques were used to examine the effects of shock wave application on coated titanium discs. Cytotoxicity was measured using a fibroblast proliferation assay. The anti-infective effect was assessed by monitoring the growth curves of three bacterial strains and by conventional culture. Microscopic analysis confirmed surface disruption of the coatings, with a complete release of silver in the focus area after shock wave application. Spectrometry detected an increase in silver concentration in the surrounding of the discs that surpassed the minimum inhibitory concentration (MIC) for both *S. epidermidis* RP62A and *E. coli* ATCC 25922. The released silver demonstrated an anti-infective effect, significantly inhibiting bacterial growth, especially at 6% and 8% silver concentrations. Cytotoxicity testing showed decreasing fibroblast viability with increasing silver concentration in the coating, with 6% silver maintaining viability above 25%. Compared to a commonly used electroplated silver coating on the market, the new coating demonstrated superior antimicrobial efficacy and lower cytotoxicity.

## 1. Introduction

Implant-related infections in orthopedic surgery present a significant challenge, impacting patient outcomes and healthcare resources [1]. The risk of periprosthetic joint infection (PJI) is increased for megaendoprostheses by approximately 20% [2] and can reach up to 50% after multiple surgical revisions [3]. The most common pathogens of PJI are Gram-positive bacteria, such as staphylococci, which have become increasingly resistant to various antibiotics [4,5]. This complicates the treatment of PJI, which mainly consists of surgery and systemic antibiotic treatment. Novel strategies for prophylaxis and treatment of PJI focus on implant surface modifications [6,7,8] or implant coatings with local antibiotic release [9,10,11]. However, the clinical application of anti-infective implant coatings is still very limited and has to comply with high regulatory requirements [12].

Over the last twenty years, the positive effects of silver coating on tumors and revision endoprostheses have been reported, especially in the case of PJI revision surgeries [13]. Side effects and cytotoxicity competing with the anti-infective effect are still debatable issues. In addition, the time of anti-infective efficacy, release kinetics, or mechanisms of inactivation are not well understood [14].

Bioabsorbable polymers loaded with antimicrobials could help overcome some of these hurdles. The challenge here is to choose a suitable biocompatible polymer that allows for the controlled release of anti-infective substances.

A novel anti-infective coating must meet specific criteria to enhance its clinical application. Firstly, it should adhere permanently to the prosthesis while preserving its original physical and mechanical properties. Secondly, it must strike a delicate balance between being effective against microorganisms without causing significant harm to native cells. Ideally, the development of resistance to anti-infective substances in the coating should be minimized. Lastly, the coating should allow for a controlled release of the anti-infective substance, which can be adjusted when needed, eliminating the requirement for additional surgical procedures. To meet these requirements, a novel anti-infective coating based on biopolymers was developed, which releases anti-infective silver into the surroundings of the implant through the application of non-invasive extracorporeal shock waves. Shock waves are acoustic waves that are defined by a rapid pressure increase within nanoseconds, followed by a low-amplitude traction phase that lasts for a few microseconds [15]. Since the 1980s, shock waves have been utilized in various medical applications, starting with the fragmentation of kidney stones and gallstones [16,17,18,19] and extending to the treatment of nonunions and enthesopathies [20,21,22,23]. More recently, there have been experimental endeavors to combat bacterial biofilms using shock wave therapy [24,25]. However, the activation of burst-release of anti-infective agents from an implant coating has not yet been reported in the literature.

The aim of the present work was to determine the appropriate silver loading to provide a local anti-infective effect with the lowest possible cytotoxicity after release via extracorporeal shock waves.

## 2. Materials and Methods

### 2.1. Coating Production

The samples were made from titanium alloy discs Ti6Al4V Grade 23 with a diameter of 14 mm, a thickness of 1.5 mm, and a centric hole of 2 mm. The number of specimens per coating and test is shown in Table 1.

The samples were sterilized by immersion in 70% ethanol. The surface of the discs was prepared by etching in 0.5 M oxalic acid (6.3 g/100 mL H_2_O) at 100 °C for 45 min and rinsing with 0.5 M calcium lactate solution after cooling to room temperature to improve the adhesion of the coating.

The coating was prepared by manual immersion in a 10% solution of poly-L-lactide (PLLA, RESOMER L 206 S, Evonik Health Care, Darmstadt, Germany) and chloroform. To 1 mL of this solution, 25 µL of silver ions were added at different concentrations (2, 4, 6, and 8%). After immersion, the discs were air-dried in a hanging position until complete evaporation of the chloroform.

### 2.2. Activation of Silver Burst Release by Extracorporeal Shock Wave Application

A clinical shock wave generator (Duolith SD1 “ultra”, Storz Medical AG, Tägerwilen, Switzerland) was used to partially disrupt the coating and to release the silver ions.

The physical principles of shock wave propagation and surface disruption are complex. In short, the shock wave propagates almost lossless through a medium *v*_1_ until it meets an interface to a medium *v*_2_ whose acoustic velocity *c* and density *ρ*, i.e., acoustic impedance (*Z* = *c* × *ρ*) differs [26,27]. Depending on the difference in acoustic impedance, the amplitude of the shock wave is divided into a reflecting and a transmitting portion. Considering plane interfaces, the reflected portion, which is associated with the pressure generation on the interface, can be determined by the reflection coefficient *Γ* [28]:

*Γ* = (*Z_v_*_2_ − *ρ _v_*_1_)/(*Z_v_*_2_ + *Z_v_*_1_). (1)

while the reflection coefficient describes the amplitude ratio between reflected and incident wave, the energy transfer at a plane interface can be calculated using the reflectance *R* [28]:

*R* = *Γ*^2^. (2)

An overview of the physical parameters of the materials used in the test set-up for shock wave activation is shown in Table 2. In the clinical setting, the shock wave propagates through the skin and soft tissues until it reaches the surface of the endoprosthesis. Due to the acoustic properties of the materials involved, the majority of the energy is released on the surface of the endoprosthesis, and thus the coating can be disrupted, and active substances can be released.

The samples were vacuum sealed in sterile bags (SteriBags, Bürkle GmbH, Bad Bellingen, Germany) with 0.5 mL aqua (B. Braun SE, Melsungen, Germany). These bags were placed in the shock wave experimental set-up surrounded by water at 37 °C, as described previously [32]. The transmission of the shock wave in water can be compared to that in soft tissue, owing to the consistency in the acoustic impedance of water and soft tissue [33]. One side of the samples was exposed to 4000 impulses (1000 impulses per quadrant). The parameters were set to an energy flux density of 1.24 mJ/mm^2^ at a frequency of 3 Hz, resulting in a total energy input of 40.55 J per sample. Using Equation (2) and considering the reflectance *R* of all interfaces in between the shock wave generator and test specimen results in an energy of 39.81 J (98.17%) reaching the PLLA surface.

After the application of the shock waves, the surrounding fluid was transferred under sterile conditions from the SteriBag into an Eppendorf tube (Eppendorf SE, Hamburg, Germany) for further analysis.

### 2.3. Determination of Silver Concentration

For the determination of the silver ion concentration, the fluid after the shock wave was transferred to a perfluoroalkoxy (PFA) vessel (AHF-Analysetechnik, Tübingen, Germany), and 100 µL of HNO_3_ (0.16 mol/L) was added to incubate overnight at room temperature.

Prior to measurement, the vessels were incubated at 70 °C for 90 min and then diluted 1:10 with H_2_O. Measurement was performed with 20 µL in triplicate by graphite furnace atomic absorption spectrometry (GF-AAS) (AAS-6300, Shimadzu, Kyoto, Japan) using the following parameters: lamp current 12 mA; wavelength 328.1 nm; BGC-D2 mode; slit width 0.7 nm. The silver standard solutions of 0, 0.5, 1, 2, and 4 µg/L Ag^+^ (Merck KGaA, Darmstadt, Germany) were used for calibration. The detection limit of the spectrometer for silver ions is 5 ng/L.

The minimum inhibitory concentration (MIC) was determined for *Staphylococcus epidermidis* RP62A (ATCC-35984, ATCC* via LGC Standards GmbH, Wesel, Germany) and *Escherichia coli* ATCC 25922 (ATCC* via LGC Standards GmbH, Wesel, Germany) using the method by Wiegand et al. (2008) [34]. For this purpose, overnight cultures with tryptic soy broth as medium were selected to prepare the bacterial suspension with a CFU count of approximately 5.0 × 10^3^ CFU/mL and broth microdilution for antimicrobial testing. The minimum bactericidal concentration (MBC) for the *E. coli* strain was also determined by plating the suspension on blood agar of all wells that had no visible turbidity. Any growth would indicate that the bacteria were only inhibited, whereas no growth would indicate a bactericidal effect.

### 2.4. Microscopic Analysis

In addition to the microbiological analysis of the supernatant, the effect of shock wave application was investigated using confocal laser scanning microscopy (CLSM) and scanning electron microscopy (SEM), including energy dispersive X-ray spectroscopy (EDS). A CLSM (VK-X3000, KEYENCE Germany GmbH, Neu-Isenburg, Germany) with a 20× lens was utilized to contactless and quantitatively analyze the disrupted coating in the sub-nanometer range. For this purpose, an area of 1930 µm × 1450 µm was recorded from each quadrant of a sample via laser scanning confocal mode and further processed in the microscope software. Subsequently, the roughness of the disrupted surface was evaluated via the 3D roughness parameter *S*_a_ using an S-filter of 2.5 µm and an L-filter of 0.8 mm in accordance with the ISO 25178-2:2021 and ISO 25178-3:2012 standards [35,36].

The SEM (Zeiss EVO MA10, Carl Zeiss Microscopy GmbH, Jena, Germany) was used for qualitative analysis of the disrupted surface area of the samples. Prior to analysis, the samples were placed on aluminum stubs via carbon adhesive discs, and a thin film of gold was deposited by DC sputtering (Polaron E5000, Polaron Equipment Ltd., Watford, UK) for 45 s at a current of 10 mA to ensure electrical conductivity. For image acquisition, an acceleration voltage EV = 5 kV, working distance WD = 11 mm, and magnifications of 100× and 200× were utilized in high vacuum mode.

In addition, an EDS (XFLASH 6|10 Detector, Bruker Co., Billerica, MA, USA) was used to analyze the samples by measuring the silver (Ag) and titanium (Ti) concentration inside and outside the activated area. The working distance was altered to WD = 10 mm to improve the signal-to-noise ratio of the emitted X-rays in this analysis. Furthermore, the acceleration voltage was increased to EV = 10 kV to ensure reliable detection of the elements Ag and Ti.

### 2.5. Cytotoxicity

The WST-1 cell proliferation assay was used to measure cytotoxicity. Primary adult human fibroblasts (PromoCell GmbH, Heidelberg, Germany) were grown in Dulbecco’s modified Eagle’s medium (DMEM) with 10% fetal calf serum (FCS), 2 mM L-glutamine and 1% antibiotic-antimycotic in 12-well plates at 37 °C and 5% CO_2_.

For the test, the medium was changed to 1100 µL DMEM with 5% FCS and 120 µL WST-1 was added. The optical density (OD) of the formazan dye formed was measured in a spectrophotometer (BMG Labtech FLUOstar Optima Fluorimeter, Ortenberg, Germany) at a wavelength of 450 nm. Viability, expressed as survival fraction, was calculated by dividing the OD of the tested samples by the OD of a control without sample at the same wavelength:

Survival fraction [%] = (OD_sample_/OD_control_) × 100. (3)

### 2.6. Anti-Infective Effect

To assess the anti-infective effect of the fluid around the disc after shock wave application, the growth curves of three different bacterial strains (*Staphylococcus aureus* 6850 (R.A. Proctor, Departments of Medical Microbiology and Immunology and Medicine, University of Wisconsin School of Medicine and Public Health, Madison, WI, USA), *Staphylococcus epidermidis* RP62A, and *Escherichia coli* ATCC 25922) were monitored. The strains were grown overnight in Tryptic Soy Broth and then adjusted to OD578 for *Staphylococci*/OD600 for *E. coli* = 0.01 and further diluted 1:10, finally reaching a bacterial load of 10^4^ colony-forming units (CFU)/mL.

In a 96-well plate, 100 µL of the solution was combined with 10 µL of the surrounding fluid collected after applying the shock waves. The OD at 575 nm (for staphylococci) or 600 nm (for *E. coli*) was measured using the Synergy HTX Multi Mode Reader (BioTek Instruments GmbH, Bad Friedrichshall, Germany) every hour for 24 h. In addition, after 3 h, bacterial suspensions were plated in serial dilutions on blood agar plates and incubated overnight at 37 °C to determine the number of colony-forming units (CFU).

### 2.7. Statistics

Statistical analysis and graphing of the results were performed using GraphPad Prism Version 9 (GraphPad Software, Inc., Boston, MA, USA). The Shapiro–Wilk test was used to assess normality. Silver concentrations and CFU count after 3 h were statistically analyzed using the Kruskal–Wallis test with Dunn’s correction for multiple comparisons. WST-1 results were tested using one-way repeated measures ANOVA followed by Tukey’s correction for multiple comparisons. Growth curves were evaluated using Friedman’s tests with Dunn’s correction for multiple comparisons. A result was considered significant with a *p*-value less than 0.05 (*), very significant with a *p*-value less than 0.01 (**), and highly significant with a *p*-value less than 0.001 (***).

## 3. Results

### 3.1. Silver Release by Shock Wave Activation

**Figure 1:**
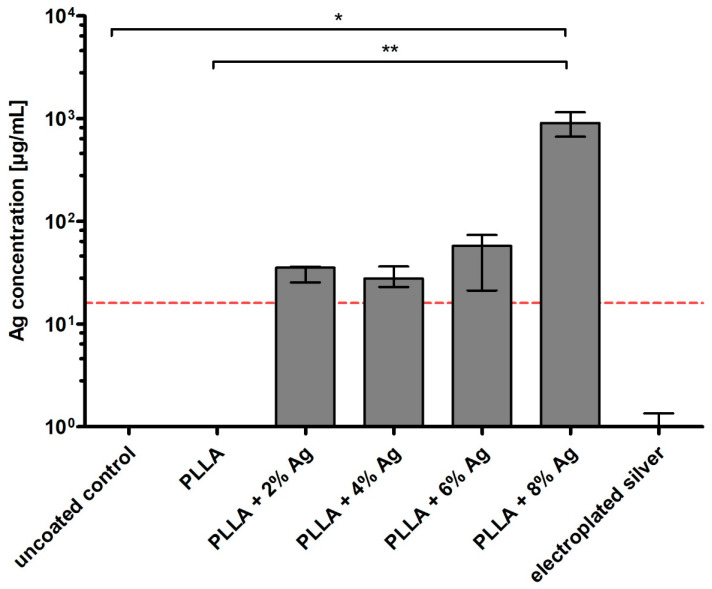
Silver concentrations (µg/mL) in the supernatant after shock wave application on uncoated control, discs with increasing concentration of Ag in the PLLA coating, and a commercially available electroplated silver coating. The red dashed line indicates the minimum inhibitory concentration (MIC) of AgNO_3_ for *S. epidermidis* RP62A and *E. coli* ATCC 25922. Bars indicate median, error bars indicate interquartile range, * *p* < 0.05 and ** *p* < 0.01 from Kruskal–Wallis test.

After the application of shock waves onto the discs, the silver concentration was determined in the surrounding fluid (Figure 1). All silver-containing PLLA coatings showed a detectable increase in silver in the surrounding fluid. All silver concentrations surpassed the minimum inhibitory concentration (MIC) for both *S. epidermidis* RP62A and *E. coli* ATCC 25922, which was determined at 16 µg/mL. Furthermore, the minimum bactericidal concentration (MBC) for this particular strain of *E. coli* was the same as the MIC, indicating that all levels of silver concentration exceeded the MBC as well. The detectable silver concentration in the surrounding fluid increased proportionally with higher silver concentrations in the coating after ESWT. Notably, the 8% Ag coating demonstrated a statistically significant difference compared to the control samples (vs. Ti6Al4V: *p* < 0.05; vs. PLLA without silver: *p* < 0.01). As expected, the uncoated control disc (Ti6Al4V) and the pure PLLA showed no detectable silver in the fluid. After applying shock waves to the already commercially available electroplated silver coating, the concentration of silver in the fluid was extremely low.

### 3.2. Microscopic Analysis

**Figure 2:**
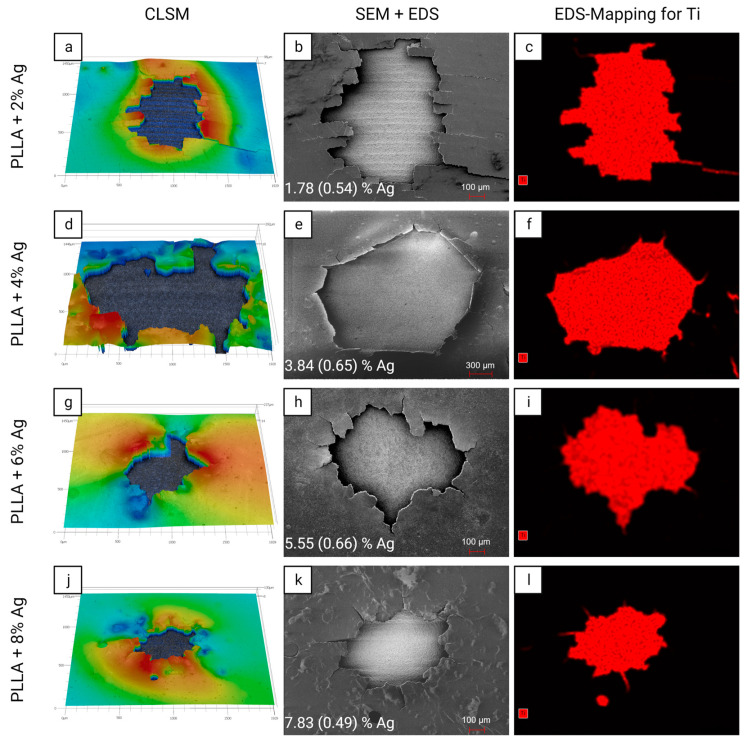
Microscopic analysis of the effect of shock wave treatment on the PLLA coatings containing silver via confocal laser scanning microscope (CLSM), scanning electron microscope (SEM), and energy dispersive X-ray spectroscopy (EDS) in one quadrant. While CLSM measurements (**a**,**d**,**g**,**j**) quantitatively confirmed a surface disruption, EDS-mappings for Titanium (**c**,**f**,**i**,**l**) demonstrated that the Ti6Al4V surface was completely exposed in the activated region. EDS measurements of Ag concentration (**b**,**e**,**h**,**k**) confirmed that the targeted silver concentration was present in all coatings. The measured Ag concentration is presented as mean (SD).

The silver release from the PLLA coating via shock wave application was analyzed with CLSM, SEM, and EDS. The results of the analyses for PLLA coatings with silver concentrations of 2%, 4%, 6%, and 8% are illustrated in Figure 2.

All coatings showed a surface disruption in each quadrant subjected to shock waves. The coating near the disrupted area was delaminated and slightly lifted. The topographic analysis of the focus area yielded a roughness of *S*_a_ = 0.646 (0.033) µm, which corresponds to the roughness of the uncoated Ti6Al4V discs of *S*_a_ = 0.665 (0.031) µm, indicating a complete release in the focus area. In comparison, the roughness of the coating before activation by shock waves was 0.074 (0.001) µm.

Further investigation of the activated areas of the samples via SEM and EDS enabled both electron-optical and chemical analysis. First, the SEM micrographs confirmed the observation of coating disruption with polygonal edges via CLSM (Figure 2b,e,h,k). The EDS analysis indicated that the silver concentration in the intact coating corresponds to the intended concentrations. In addition, the EDS-mapping for the element Titanium (Ti) in the right column of Figure 2c,f,i,l confirmed a complete disruption and release of the coating from this focus area. The size of the disrupted area was independent of the silver concentration in the PLLA with a mean (SD) size per quadrant of 0.9505 (0.4351) mm^2^.

### 3.3. Cytotoxicity

**Figure 3:**
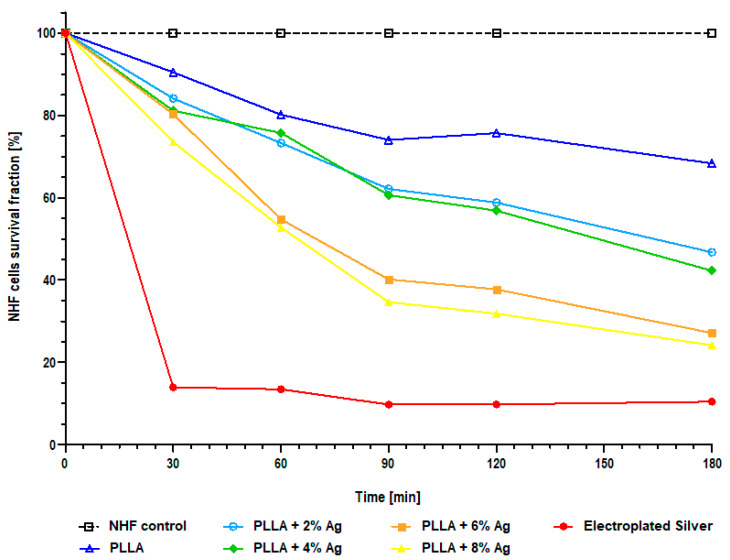
NHF cell viability expressed as survival fraction using water-soluble tetrazolium salt (WST-1) assay with different coatings. Cell viability decreases as the silver concentration of the coating increases.

The cytotoxicity of the supernatant against normal human fibroblasts (NHF) was tested using the water-soluble tetrazolium salt (WST-1) cell viability assay. The viability calculated from the measured absorbance over time is shown in Figure 3.

The fibroblast viability was highest for the pure PLLA coating. However, as the silver concentration in the coating increases, the viability progressively decreases. Notably, the coating containing 8% Ag showed the lowest viability, with less than 25% viability observed after 180 min. On the other hand, the coating with 6% Ag maintained viability above the 25% threshold over the same duration. Importantly, it is worth emphasizing that all the coating variants demonstrated superior cell viability compared to the electroplated silver coating that has been clinically used for several years.

### 3.4. Anti-Infective Effect

To assess the anti-infective effect of the released silver, bacterial growth was determined with optical density for *Staphylococcus aureus*, *Escherichia coli,* and *Staphylococcus epidermidis*. In addition, conventional bacterial culture was performed for *S. aureus* and *E. coli* after 3 h.

**Figure 4:**
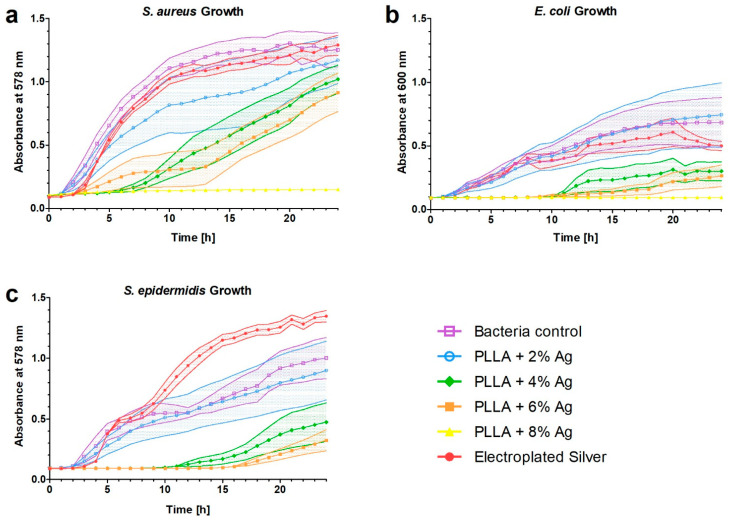
Growth curves of bacteria incubated with surrounding fluid after shock wave application on different coatings. (**a**) *Staphylococcus aureus*. PLLA + 8% Ag showed a complete prevention of growth over 24 h, while 6% and 4% showed a delay of 10 h. (**b**) *Escherichia coli*. Growth inhibition over 24 h is present at 8% and 6% silver, and growth delay for 11 h is present at 4% silver. (**c**) *Staphylococcus epidermidis*. Growth is delayed for 12 h for the 4% and 6% coatings.

Figure 4 illustrates the bacterial growth within the surrounding fluid of PLLA coatings with varying concentrations of silver after shock waves. Increasing silver concentration in the coating proved to be a more effective inhibition of bacterial growth for all three strains. The coatings containing 6% and 8% Ag demonstrated a significant reduction in growth compared to the controls and the electroplated silver coating (*p* < 0.001). Remarkably, PLLA + 8% Ag exhibited complete inhibition of bacterial growth for 24 h. PLLA coatings with 6% and 4% Ag delayed growth for approximately 10 h, with the least efficacy observed against *S. aureus*. Notably, all PLLA coatings showed significantly higher inhibition of bacterial growth of *S. aureus* compared to the electroplated silver coating (electroplated silver vs. PLLA 4% Ag: *p* < 0.01; vs. PLLA 6% Ag: *p* < 0.001; vs. PLLA 8% Ag: *p* < 0.001). The growth of *E. coli* was not inhibited by 2% Ag and outgrew the control after 19 h. Apart from the 8% Ag coating, the 6% Ag coating also inhibited the growth of *E. coli*. The 4% Ag coating, on the other hand, delayed growth for a significant period of 11 h. As for *S. epidermidis*, both the 4% and 6% Ag coatings inhibited growth for 12 h, whereas the 2% Ag coating had no effect on growth.

**Figure 5:**
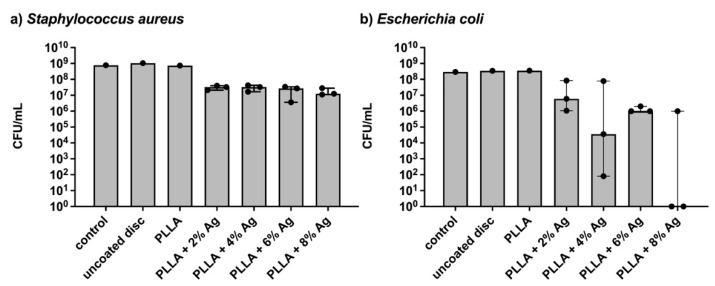
Colony Forming Units (CFU) per ml of *Staphylococcus aureus* (**a**) and *Escherichia coli* (**b**) following a three-hour incubation at 37 °C. The samples include a control group without intervention, uncoated titanium discs, and coated discs with increasing silver concentrations exposed to shock waves. The bars indicate the median and the error bars indicate the interquartile range.

After three hours of incubating the bacterial cultures in the surrounding fluid, the number of *S. aureus* colony-forming units (CFU) showed a remarkable reduction by a factor of ten to twenty for the PLLA + Ag coatings compared to the three control samples (Figure 5). The CFU counts of *E. coli* were also inhibited by all PLLA + Ag coatings, though the results exhibited more variability. The CFU counts for the 2% and 4% Ag coatings were reduced by ten- to fortyfold, respectively, while the 6% Ag coating resulted in a twenty- to thirtyfold reduction. Notably, two out of the three samples with 8% Ag were completely inhibited, whereas one sample’s CFU count was reduced by a factor of two to ten.

## 4. Discussion

### 4.1. Silver Release by Shock Wave Activation

After the application of shock waves, the released silver content in the surrounding fluid was measured. The findings indicate that higher silver concentrations in the coating result in increased silver release. In a prior publication, we were able to demonstrate within an in vitro experiment set up over a duration of four days that the quantity of silver released from the PLLA coating, in the absence of shock wave application, remained negligibly minimal [32]. Therefore, the application of shock waves enables controlled release with a defined silver content in the biopolymer. In contrast, the electroplated silver coating exhibited significantly lower silver content in the surrounding fluid. In the literature, varying minimum inhibitory concentrations (MICs) for cocci bacterial species are reported [37]. However, the measured values in the supernatant for the corresponding silver concentrations in the coating surpassed the MIC for both *S. epidermidis* RP62A and *E. coli* ATCC 25922 by at least 11 µg/mL. Additionally, the minimum bactericidal concentration (MBC) for the *E. coli* strain was equal to its MIC, which was also surpassed by the silver concentrations. Notably, shock waves applied to the electroplated coating failed to release relevant amounts of silver ions from the surface.

### 4.2. Microscopic Analysis

Through microscopic analysis via CLSM, it was observed that the coating exhibited bulging in the immediate vicinity of the activated area. It is assumed to be caused by the reflection of the shock wave when it impacts the titanium surface (reflection coefficient *Γ* = 75.76%). The irregular and angular shape of the activated region, which did not align with the focal zone of the shock wave device, suggested that the disrupted area had not been removed in a single piece. Instead, it appeared that a series of impulses, which cannot be quantified yet, led to the gradual disruption of small areas at a time, resulting in the successive removal of numerous small PLLA fragments. The accompanying increase in the surface/volume ratio of those PLLA fragments leads to more efficient drug elution. Through SEM and EDS analysis, it was confirmed that the silver concentration in the actual coating matched the intended levels. EDS Mapping further demonstrated that the silver in the focus area could be entirely released from the surface and distributed into the surrounding environment. This mechanism offers considerable advantages, as it enables anti-infective agents to exert their effects not only on the coated surfaces but also in the proximity of the implant. For large endoprostheses, certain areas like gaps between connecting modules, articulating surfaces, and regions with direct bone contact are not intended to be coated. This method ensures that the agents can target these specific areas effectively without the need for direct coating.

### 4.3. Cytotoxicity

Cytotoxicity of the coating was tested with a WST-1 cell viability assay. The results indicated that cytotoxicity increased with higher concentrations of silver in the coating. In a previous study by Schulze et al. [32], the cell viability of the clinically used electroplated silver coating mostly remained below 25% compared to the control. In this particular application, maintaining sufficiently high cell viability is of utmost importance. Joints that have undergone multiple revisions often exhibit scar tissue, which inherently has limited healing potential. Thus, the use of the coating becomes even more critical to support optimal cell viability and enhance the potential for successful healing in such cases. Therefore, the objective of this new coating was to achieve better results, ensuring cell viability above 25%.

Among the tested coatings, only the one with 8% silver falls below the set viability limit after three hours. On the other hand, the 6% Ag coating successfully achieves the goal of maintaining fibroblast viability above 25% while also demonstrating strong anti-infective effects. Based on these experiments, the 6% silver concentration appears to be the most suitable choice.

Given the potential risk that the coating might lead to compromised osseointegration when in direct bone contact, as observed in clinical studies involving other surface modifications [38], this coating is intended solely for regions of the prosthesis not in direct contact with bone. In the case of modular mega-endoprostheses, this pertains to the modular components situated between the articulating components and the bone-anchored stem.

The silver nitrate is enclosed within the poly-L-lactide (PLLA) that gradually resorbs, resulting in only the top layer exposing a constant 6% Ag concentration to the surrounding soft tissues. Hence, a thicker coating should not pose an elevated risk. PLLA was selected due to its biodegradable nature and slow degradation time, ensuring a gradual release of silver [39]. The application of shock waves offers an additional advantage by significantly increasing the silver content in the proximity, allowing for rapid bacterial elimination when required.

### 4.4. Anti-Infective Effect

The anti-infective effect was tested using bacterial growth curves of *S. aureus*, *E. coli,* and *S. epidermidis*. The effect showed a dose-dependent manner. The coating with the 2% silver concentration did not demonstrate a satisfactory effect on the growth of the bacteria. The higher doses inhibited the growth of all bacteria for a certain period. The anti-infective effect on *S. aureus* and *E. coli* was evident in conventional culture plating after 3 h. It is evident from both assessments that the effect is more pronounced on *E. coli.*

Silver ions act in two ways on the bacteria. Firstly, they bind to the murein wall and alter its permeability. This feature ensures that a greater anti-infective effect is seen in Gram-negative bacteria with their thinner murein walls [40]. Secondly, the ions bind to the thiol groups of the enzymes, inactivating them. This, in turn, disrupts the respiratory chain and the TCA cycle. Hydroxyl radicals accumulate, and the bacteria’s DNA is damaged [41]. The first mechanism can be classified as bacteriostatic, while the second is bactericidal [40]. As the latter is beneficial in reducing the likelihood of resistance, the concentration of free silver ions should be as high as possible when fighting infection.

Based on the silver measurements in the supernatant and the microbiological evaluations of the anti-infective effect, it is evident that the release and efficacy of silver increase with higher concentrations in the coating. Therefore, the highest tested concentration in the coating, which is 8%, would be advantageous. However, this coating also displayed a viability of less than 25% in normal human fibroblasts, indicating higher cytotoxicity compared to the commonly used electroplated silver coating. As a result, it was excluded from further experiments prior to the conclusion of the study. Based on the results of these in vitro experiments, the 6% coating seems to offer the best balance between cytotoxicity against human cells and anti-infective activity.

### 4.5. Limitations

As the coating manufacturing process is currently manual and performed through a dipping process, there may be certain differences and variations. A standardized coating procedure that ensures consistent quality is currently under development. It is possible that variations between different batches could explain the dispersion of results observed in Figure 5. On the other hand, SEM + EDS images demonstrate that the desired silver concentrations have been well achieved in their respective coatings. Furthermore, the debonding characteristics caused by shock wave application in the vicinity of the activated area have not yet been studied in detail. However, surface modifications to improve the adhesion of coatings are part of the ongoing development.

The conditions in these in vitro experiments vary from those present in an in vivo or in situ environment. It is known that physiological fluids and their protein content can influence and potentially deactivate silver ions [42]. Mulley et al. demonstrated that reduced thiol groups found in compounds commonly present in the blood, like glutathione, can diminish the toxicity of silver ions by binding to them [43]. Considering this potential competition between physiological fluids and tissue cells, it is probable that the cytotoxic effect could be less pronounced when the implant coating is applied in vivo, compared to the results obtained in these in vitro experiments.

This could also be the reason why, despite the relatively high cytotoxicity observed in the in vitro experiment, the commercially available electroplated silver coating has not shown significant issues with endoprosthesis integration and wound healing in clinical application. However, it is also quite possible that this effect leads to a reduced anti-infective efficacy against bacteria. Future studies must, therefore, attempt to replicate the physiological conditions of the surrounding fluids as closely as possible. Additionally, the immune system plays a role, aiding in the battle against infection but also possibly triggering a foreign body reaction. In vivo experiments are necessary to examine these effects in detail.

## 5. Conclusions

This study introduces a novel biopolymer coating containing silver nitrate. The results demonstrate that it is feasible to achieve controlled on-demand release of silver using extracorporeal shock waves. Silver can be entirely released in the focus area from the surface into the surrounding environment.

In the clinical scenario, silver ions could be released from an endoprosthesis surface whenever needed, exerting their effects against virtually all microorganisms that can cause periprosthetic infections. In addition to purely passive surface coatings designed to prevent bacterial biofilm formation, the release of very high concentrations of anti-infective substances can also occur in the surroundings of the endoprosthesis. This approach could be of practical benefit in the treatment of periprosthetic joint infections.

At all tested silver concentrations, the cytotoxicity of this novel coating is significantly lower than that of a traditional electroplated silver coating. Moreover, the anti-infective effect of the silver-loaded coating improves with higher silver concentrations, and it remains more effective than the traditional electroplated silver coating when evaluated in vitro.

An ideal silver concentration in the coating, based on in vitro experiments, appears to be 6%, as it exhibited a strong anti-infective effect with acceptable cytotoxicity. Further preclinical studies should, therefore, be conducted evaluating this concentration.

## 6. Patents

A patent application has been filed for the coating (international publication number: WO2023025944).

## Figures and Tables

**Table 1:** Matrix of specimens per test.

	Ag Concentration	WST-1	*S. aureus* Growth Curve	*E. coli* Growth Curve	*S. epidermidis* Growth Curve	CFU (3 h)
Ti6Al4V	2	–	–	–	–	1
PLLA	5	6	–	–	–	1
PLLA + 2% Ag	5	6	6	6	6	3
PLLA + 4% Ag	4	6	10	10	8	3
PLLA + 6% Ag	6	6	8	8	6	3
PLLA + 8% Ag	3	6	1	1	–	3
Electroplated Silver	2	1	2	2	2	–

**Table 2:** Physical parameters of shock wave energy release on different materials.

Medium *v*	Density *ρ* [kg/m^3^]	Acoustic Velocity *c* [m/s]	Impedance *Z* [Ns/m^3^]
Water	998	1483	1.48 × 10^6^
LDPE	920	1950	1.79 × 10^6^
PLLA	1320	2319	3.06 × 10^6^
Ti6Al4V	4450	4987	1.83 × 10^7^

LDPE: Low-density polyethylene; PLLA: Poly-L-lactic acid; Ti6Al4V: Titanium alloy with 6% aluminum and 4% vanadium; Material constants from [29,30,31].

## Data Availability

The data presented in this study are available on request from the corresponding author. The data are not publicly available due to privacy restrictions.
